# Incorporating the patient-centered approach into clinical practice helps improve quality of care in cases of hypertension: a retrospective cohort study

**DOI:** 10.1186/s12875-020-01183-0

**Published:** 2020-06-12

**Authors:** Nida Buawangpong, Kanokporn Pinyopornpanish, Wichuda Jiraporncharoen, Nisachol Dejkriengkraikul, Pakorn Sagulkoo, Chanapat Pateekhum, Chaisiri Angkurawaranon

**Affiliations:** 1 Department of Family Medicine, Faculty of Medicine, Chiang Mai University, 110 Inthawarorot Rd., Sriphum, Muang, Chiang Mai, 50200 Thailand; 2 Department of Biochemistry, Faculty of Medicine, Chiang Mai University, 110 Inthawarorot Rd., Sriphum, Muang, Chiang Mai, 50200 Thailand

**Keywords:** Primary health care, Family medicine, Patient-centered medicine, Hypertension, Guidelines, Clinical practice

## Abstract

**Background:**

Treating hypertensive patients by integrating the patient-centered approach would influence the practice and outcome of treatment. Our purpose was to determine whether the implementation of a patient-centered approach in health care delivery can improve adhering to guidelines and the quality-of-care.

**Methods:**

A retrospective study was conducted using secondary data from the electronic medical records of the patients treated in the two primary care outpatient settings at the Family Medicine (FM) and Social Security (SS) clinics. A key feature of the FM clinic is the incorporation of a patient-centered approach in its service delivery. Individual information regarding initial assessment and treatment at the follow-up visits was reviewed for 1 year. Comparison of adherence to treatment guidelines between the two primary care clinics was performed by using chi-square, Fisher’s exact test or a t-test. To explore the difference in blood pressure and BP control between the two clinics, linear and logistic regression analysis respectively were performed with an adjustment for CV risk score in 2016 as a key confounder.

**Results:**

The evidence included 100 records from each clinic, showed variation between the two primary care sites. The FM clinic had more complete records regarding family history of hypertension, assessment for secondary causes, prescription for lifestyle modification and appropriate adjustment of medication. Higher levels of blood pressure control were recorded in the FM clinic, specifically systolic pressure 2.92 mmHg (p = 0.073) and diastolic pressure 5.38 mmHg (*p* <  0.001) lower than those recorded in the SS clinic. There was a 2.96 times higher chance for BP goals to be achieved in patients in receipt of hypertensive care at the FM clinic (*p* = 0.004).

**Conclusions:**

Adopting a patient-centered approach in service delivery could improve the quality of care for hypertension patients in primary care in Thailand.

## Background

Hypertension is one of the leading causes of mortality worldwide and the number of patients is increasing [1, 2]. Uncontrolled blood pressure (BP) leads to the serious complications such as cardiovascular diseases [3]. It has been found that BP in 35.8% of patients in Thailand cannot be controlled [4].

Treating hypertensive patients by complying with standard treatment guidelines would create more BP controlled [5–8], and consequently reduce the morbidity and mortality [9, 10]. However, there might be some barriers preventing physicians from adhering strictly to treatment guidelines, including the attitude and practice of the physician, complexity of recommendations, and the actual setting (timing and availability of facilities and tools) [11]. Furthermore, in order to achieve the treatment goals, it is not only how the physicians treat, but also how the patient gets involved in the management of their own health care process [12].

Many hypertension treatment guidelines advocate a patient-centered approach to be integrated in all aspects of service care [13, 14]. The integration of a patient-centered approach into service delivery can be formalized through the concept of a patient-centered medicine (PCM) approach. The concept of PCM includes six main components [15]: exploring both the disease (biomedical aspect) and the illness experience (psychosocial aspect); understanding the whole person (context); finding common ground (for treatment); incorporating prevention and health promotion; enhancing the patient-doctor relationship; and being realistic. These six components of care promote collaboration between the doctor and patient by concerning the patient’s context, including socioeconomic status, in treatment plans and focusing on continuity of care [16–19].

Thailand has incorporated the concept of PCM into the Thai hypertension treatment guidelines since 2015. It was recommended briefly in one paragraph that physicians should concern themselves with forming individual treatment plans for the patients [10]. The formal concept of PCM has only been introduced and adopted as part of the competency in Family Medicine since the start of the training program in Thailand about 15 years ago [20].

In order to assess the potential benefits of integrating PCM in delivering healthcare to hypertensive patients, a clinical audit of medical records was carried out in two primary care clinics. The clinics were: 1) the family practice clinic run by the Department of Family Medicine (FM clinic) and 2) the general practice clinic for patients under the social security scheme (SS clinic). Both clinics are part of Maharaj Nakorn Chiang Mai Hospital**,** they have access to the same physical resources from the hospital, such as readiness of facilities and tools, capability of further assessment or medical record system. The differences between the two clinics are described in the Methods section.

The aim of this review was to explore whether adherence to hypertension treatment guidelines differs between these two different service settings, effect on ability to do PCM, and to explore whether the differences are associated with better control of hypertension.

## Methods

### Study design

This is a retrospective review of electronic medical records of patients who received hypertension treatment in FM clinic and SS clinic in Maharaj Nakorn Chiang Mai Hospital.

### Setting

FM clinic is a teaching and service clinic for medical students and family medicine residents. The service in this clinic is generally provided by Family Medicine residents and staff who are trained in the PCM approach. The number of patients per day is restricted. An appointment can be made to see the same physician for continuity of care.

The SS clinic is a clinic to serve patients who use the social security scheme. The service being provided by a rotation of the residents and staff who are trained in a variety of specialisms. Consequently, there are variations in practice between the physicians and continuity of care by the same physician is nearly impossible. Supplementary Table 1, Additional file 1 shows the details of the differences between these two clinics.

### Data collection

The sample size calculation was conducted based on the prior statistical evidence in these two clinics. The hypertension control rate of FM clinic and SS clinic were 65 and 45%, respectively. We used the two independent proportion formula. To reach a power of 80% and alpha value of 0.05, the estimated size sample was 96 people per unit of service. We then decided to take 100 samples from each clinic.

The first 100 hypertensive patients from each clinic that met the inclusion criteria were included in the review. The inclusion criteria were those who had a record of any visit in October 2016 and had records of further visits in a 1 year follow up period in September 2017. To avoid cross contamination, any records of the patients who had a consultation with a family medicine physician in the SS clinic were excluded. As about 5% of the services in SS clinic provided by family physicians which would impact the interpretation. The first 20 records of the same patients from each clinic were reviewed independently by two researchers (NB and PS). The reviewers compared their assessment and any disagreement was resolved through discussion and consensus. The next 160 records (80 for each reviewer) were randomly assigned and reviewed by either one of the two researchers.

### Adherence to treatment guidelines

The Thai guidelines for treatment of Hypertension 2015 follows the 2013 Practice guidelines for the management of arterial hypertension by the European Society of Hypertension and the European Society of Cardiology and The Sixth Report of the Joint National Committee on Prevention, Detection, Evaluation and Treatment of High Blood Pressure [10]. The researchers developed a case record form by referring to these treatment guidelines which were broken down into four broad sections:

1. Initial assessment
2. Continuous monitoring and assessment for target organ damage (TOD)
3. Prescription for lifestyle modification
4. Prescription of medication and dose adjustment

For section 1, detailed accurate initial assessment at diagnosis, evaluation of secondary hypertension, and family history assessment. For section 2, continuous monitoring and assessment for TOD records were assessed to see whether they had such investigations. For lifestyle modifications in section 3, the medical records were assessed for whether there was any documentation of lifestyle modifications. For prescription of medication in section 4, records were assessed whether the physician had adhered to the recommended dosage and class of hypertensive medication as well as whether the physician adjusted the dose of the medication when the target blood pressure was not achieved. The definition and assessment period for each topic can be found in Supplementary Table 2, Additional file 2.

### Blood pressure control

The mean BP over the year, excluding the first BP in October 2016 (as the first BP was included in cardiovascular (CV) risk assessment and treated as a confounder), was used as an outcome and also used to classify whether the control of BP was achieved. BP goals were 140/90 mmHg for general patients and 130/80 mmHg for chronic kidney disease patients with proteinuria, diabetes patients aged 50 years or less or with albuminuria.

### Statistical analysis

Comparison of adherence to treatment guidelines between the two primary care clinics was performed by using chi-square, Fisher’s exact test or a t-test. To explore the difference in blood pressure and BP control between the two clinics, linear and logistic regression analysis respectively were performed with an adjustment for CV risk score in 2016 as a key confounder. A CV risk calculation was done using a Thai CV Risk Score application which estimates the 10-year risk of developing a major cardiovascular event based on the patients age, gender, smoking behavior, presence of diabetes and their last measurement of blood pressure, total cholesterol, low-density lipoprotein cholesterol (LDL-C) and high-density lipoprotein cholesterol (HDL-C) in 2016. The software used for analysis was STATA (version 15.1).

## Results

Age, comorbidities, type of antihypertensive drug, and the CV risk between the patients attending the two clinics were statistically significant differences (described in Table 1). Patients attending the FM clinic were older than those attending the SS clinic (mean age 61.68 vs 52.14, *p* <  0.001) with a higher average percentage of 10-year CV risk (12.8 ± 7.75 vs 6.90 ± 5.28, *p* <  0.001).

**Table 1:** Characteristics of the patients in the FM and SS clinics

Characteristics	FM clinic *N* = 100	SS clinic *N* = 100	*P*-value
Female gender, n	55	65	0.149
Age (year), mean (SD)	61.68 (6.44)	52.14 (7.03)	< 0.001^a^
BMI (kg/m^2^), mean (SD)	26.10 (4.12)	26.29 (3.77)	0.738^a^
Education level^c^, n (%)			0.120
- Primary school or lower	9/30 (30.00)	27/58 (46.55)	
- High school/ Vocational school	11/30 (36.67)	22/58 (37.93)	
- Bachelor’s degree or higher	10/30 (33.33)	9/58 (15.52)	
Occupation with regular salary^c^, n (%)	41/69 (59.42)	16/65 (24.62)	< 0.001
Comorbidities			
● Diabetic mellitus type II, n	34	39	0.463
● Dyslipidemia, n	86	55	< 0.001
● Chronic kidney disease, n	8	4	0.234
● Cerebrovascular disease, n	9	0	0.003^b^
● Cardiovascular disease, n	6	0	0.029^b^
Use of anti-hypertensive drugs for 1 year	92	99	0.035^b^
Current anti-hypertensive drugs			
● Calcium channel blockers, n	62	66	0.556
● Beta blockers, n	6	24	< 0.001
● ACE inhibitors, n	9	32	< 0.001
● Angiotensin II receptor blockers, n	47	25	0.001
● Mineralocorticoid receptor antagonists, n	1	0	1.000^b^
● Diuretics, n	7	12	0.228
Active alcohol drinker^c^, n (%)	21/87 (24.0)	22/100 (22.0)	0.729
Current smoker^c^, n (%)	4/86 (4.65)	2/100 (2.00)	0.417^b^
Percentage CV risk calculation, mean (SD)	12.80 (7.75)	6.90 (5.28)	< 0.001^a^
Baseline BP (mmHg), mean (SD)			
● Systolic BP	134.96 (13.8)	137.36 (14.5)	0.232^a^
● Diastolic BP	77.76 (10.52)	82.03 (9.68)	0.003^a^
Mean BP in 1 year (mmHg), mean (SD)			
● Systolic BP	132.71 (10.7)	134.21 (10.1)	0.308^a^
● Diastolic BP	75.62 (7.21)	80.98 (7.87)	< 0.001^a^
Mean BP excluding baseline (mmHg), mean (SD)			
● Systolic BP	131.99 (11. 2)	132.90 (10.2)	0.548^a^
● Diastolic BP	74.98 (7.54)	80.47 (8.64)	< 0.001^a^

*BMI* body mass index, *BP* blood pressure

^a^ t-test

^b^ Fisher’s exact test

^c^ Evaluated among those with data in electronic records

### Initial assessment

Documentation of the initial assessment in the medical records of patients treated at the FM clinic had more complete history taking records for both family history (26% vs 2%, *p* <  0.001) and secondary causes of hypertension than those in the SS clinic (76% vs 60%, *p* = 0.015). Only about 4% of patients had documentation pertaining to the physical examination for evidence of secondary hypertension in both clinics (Table 2).

**Table 2:** Evidence of initial assessment on history taking and physical examination

Evidence of initial assessment on history taking and physical examination	FM clinic N = 100	SS clinic N = 100	*P*-value
History taking			
● Family history of hypertension, n	26	2	< 0.001^a^
● Assessed for secondary hypertension, n	76	60	0.015
Physical examination			
● Evidence of secondary hypertension, n	3	5	0.470

^a^ Fisher’s exact test

### Continuous monitoring and assessment for TOD

The evidence of history taking of hypertension related symptoms or TOD, and risk factors was more complete in the FM clinic. Both clinics monitored blood pressure at a high rate. However, calculation of body mass index was documented in only 24% of patients attending the FM clinic and in only 2% of patients attending the SS clinic (*p* <  0.001). Most patients (> 90%) had their glucose, lipid profiles and kidney function monitored in both clinics (Table 3).

**Table 3:** Evidence of continuous monitoring and assessment for target organ damage

Topics	FM clinic *N* = 100	SS clinic *N* = 100	*P*-value
History taking			
● History of hypertension, n	96	78	0.001^a^
● Risk factors, n	92	99	0.035^a^
● Symptoms suggestive of TOD, n	85	65	0.001
Physical examination			
● BP and PR measurement, n	98	100	0.497^a^
● BMI calculation and WC measurement, n	24	2	< 0.001^a^
● TOD and cardiovascular disease, n	85	45	< 0.001
Laboratory investigation			
● Fasting plasma glucose, n	93	99	0.065^*^
● Serum TC, HDL-C, LDL-C, triglyceride, n	100	100	N/A
● Serum electrolytes, Cr, GFR calculation, n	94	99	0.118^a^
● Hemoglobin or hematocrit, n	43	43	1.000
● Urinalysis, n	65	59	0.382
● Electrocardiography, n	28	33	0.443
● CV risk assessment at OPD, n	3	1	0.621^a^

*BMI* body mass index, *BP* blood pressure, *Cr* creatinine, *CV risk* cardiovascular risk, *GFR* glomerular filtration rate, *HDL-C* high-density lipoprotein cholesterol, *LDL-C* low-density lipoprotein cholesterol, *PR* pulse rate, *TC* total cholesterol, *TOD* target organ damage, *WC* waist circumference, *OPD* outpatient clinic

^a^ Fisher’s exact test

### Prescription for lifestyle modification

Prescription for lifestyle modification was not commonly recorded. Details are described in Table 4. However, it is noted that it was more commonly recorded in the FM clinic. Advice given on exercise (51% FM clinic vs 17% SS clinic, *p* <  0.001) and dietary approach (46% FM clinic vs 27% SS clinic, p = 0.005) was of statistically significantly higher incidence at the FM clinic.

**Table 4:** Evidence of giving advice on lifestyle modification

Topics	FM clinic *N* = 100	SS clinic *N* = 100	*P*-value
Weight reduction, n	23	1	< 0.001^a^
Appropriate exercise, n	51	17	< 0.001
Dietary approach, n	46	27	0.005
Limiting of alcohol intake for drinkers, n/No. of drinkers (%)	10/21 (47.6)	12/22 (54.5)	0.663
Smoking cessation for smokers, n/No. of smokers (%)	1/4 (25.0)	1/2 (50.0)	1.000^a^

^a^ Fisher’s exact test

### Prescription of medication and dose adjustment

In case of the appropriateness of choice of type and dosage of anti-hypertensive drug treatment, the majority (over 90% of patients) received appropriate initial treatment (Table 5). There was no evidence to suggest any difference in adherence to recommended guidelines for prescription of medication between the clinics. However, patients attending the FM clinic used more fixed-dose combinations. When the BP was uncontrolled, 60.3% of patients in the FM clinic received adjustments to their mediation, comparison to 34.8% in the SS clinic (p = 0.003).

**Table 5:** Evidence of appropriateness of prescription and adjustment of anti-hypertensive medication

Topics	FM clinic *N* = 100	SS clinic *N* = 100	*P*-value
Ever used an anti-hypertensive drug during the last year, n	92	99	0.035^a^
Starting with appropriate dosage of medication, n (%)	91/92 (98.9)	99/99 (100)	0.482^a^
Use of an appropriate class at the start, n (%)	88/92 (95.6)	94/99 (94.9)	1.000^a^
Choice of the most appropriate medication for the specific conditions, n (%)	79/92 (85.9)	88/99 (88.9)	0.663
Never used ACEIs together with ARBs, n/No. of patients using more than one type of medication (%)	34/34 (100)	47/48 (97.9)	1.000^a^
Prescription of fixed-dose combinations, n/ No. of patients using more than one type of medication (%)	12/34 (35.3)	3/48 (6.25)	0.001
Adjustment of dosage of medication when blood pressure goal not achieved, n/No. of patients who had ever had uncontrolled BP (%)	38/63 (60.3)	24/69 (34.8)	0.003

^a^ Fisher’s exact test

### Blood pressure control

Comparison of BP control between the clinics by adjusting for the calculated CV risk score, indicates there is some weak evidence to suggest that the average BP was lower among patients receiving care at the FM clinic. The average systolic blood pressure was 2.92 mmHg lower among those attending the FM clinic (95% CI − 6.12 to 0.28, p = 0.073) and the average diastolic blood pressure was 5.38 mmHg lower (95% CI − 7.89 to - 2.87, *p* <  0.001) than those attending the SS clinic. After adjustment for CV risk score, patients attending the family medicine clinic were almost 3 times more likely to have a controlled average BP (OR 2.96, 95% CI 1.41 to 6.22, *p* = 0.004).

## Discussion

In general, at both clinics, there was good adherence to the clinical guidelines for initial assessment, continuous monitoring, assessment for TOD and correct initiation of medication. Documentation regarding prescription of lifestyle modification was low but was found more commonly among those attending the FM clinic. Patients were also more likely to receive adjustment to their medication if hypertensive control was not met at the FM clinic. Evidence suggested that the average BP was lower among patients receiving care at the FM clinic.

Analysis of history taking showed that history about family members experiencing hypertension was noted more by family physician. The PCM approach adopted by family physicians focuses on the patient context which includes a family-oriented approach. This issue might not be regularly assessed as it is not commonly used in making a plan for treatment. Shared behaviors among family members may affect patient’s behaviors. The physician may use knowledge regarding the family’s influence on behaviors in the counseling process. In addition, family support has been identified as one of the facilitators for patient’s hypertension self-management [21]. A Family-oriented approach and enhancing health literacy to the family members could be beneficial in caring hypertension patients.

For physical examination and treatment planning, measurement of body mass index (BMI) and the assessment of 10-year CV risk were similarly low in both clinics. Both of these aspects are useful for raising of awareness in patients [22–24] and their physicians and enhance the efficacy of treatment [10, 25–28]. Technology could be used to help fill in the indices and CV risk results prior to the examination. The results show that the number of patients screened for smoking and alcohol drinking was higher in the SS clinic as it routinely done by the nurses before seeing the physician following the standard record form. This suggests that task shifting could be shared among the different types of care providers to help improve adherence to clinical guidelines [29]. Moreover, using assistive tools such as a well-developed order set could help improve compliance for assessment and management [30].

Appropriate adjustment of the dose and discussing on changing risky behaviors are practiced more frequently in the FM clinic, may be due to the longer consultation time available which allows for PCM and the finding of workable ways forward and common ground [31]. The goal is initially set together, promoting the development of trust and a good relationship. The continuity of care with the same physician would give a higher chance of agreement on dosage modification and overall management with consent [32, 33].

The evidence concerning the measurement of the integration of PCM in practice using the routine data records is scarce [34]. However, this is concurrent with the results from prior studies that adherence to guideline is associated with better BP control [5–7]. The implementation of PCM also shows a correlation with better quality of care and patient outcomes. This is supported by the results from other countries that person-centered care in hospitalized patients can improve health outcomes [35, 36].

These findings suggest that adopting a more PCM approach in the delivery of primary care services may help improve hypertensive care in Thailand through increased adherence to standard guidelines which would subsequently improve control of BP. Any method to enhance the PCM practice should be useful. This should include concerning more about family context, collaborating with the patient in making a treatment plan and supportive service system.

### Strengths and limitations

The strength of this study was the use of the same electronic medical record system which standardized many aspects of the data. This reflects real world practice as well as being able to control importation systems and contextual factors associated with hypertensive control. However, there were some limitations. Firstly, data was derived from routine records, which might underestimate the adherence to guidelines if these practices are not documented. The necessitated faster clinical pace in the SS clinic could lead to poorer documentation. There is a need for recognition by all stakeholders that every record is important, especially in the case of continuity of treatment. All information related to disease assessment and treatment should recorded even if it only consists of a short phrase. Due to time pressures in the SS clinic the use of administrators to complete records would ease the workload. Secondly, further adjustments for SES were not possible as it’s not routinely recorded in electronic records database and the interpretation of results could be limited by the decreased sample size. Due to the small sample size and from using one clinic in each arm in a single setting, the results may not be generalizable. Further research using multiple settings would be useful. Moreover, PCM should have already been tailored to each individual socioeconomic conditions. Finally, the study was conducted under the hypothesis that PCM is integrated in the practice and services. The performance pertaining to PCM was not directly measured as our aim was not to assess the perceptions or experiences about PCM practice as conducted in most studies [34]. Limited by the retrospective nature of the design, our aim was to assess the potential benefits of integrating a patient-centered approach in delivering hypertensive care in accordance to quality indicators and practice guidelines.

## Conclusion

There is room for improvement in adherence to clinical practice guidelines for treatment of hypertension. Task shifting, use of standing orders or technology to support many aspects at the clinic would improve compliance to guidelines. Adopting a patient-centered approach, through concepts of PCM, in delivering healthcare services could also improve the quality of care for hypertensive patients in Thailand.

## Supplementary information

**Additional file 1: Table S1.** The demographic data of two primary outpatient department clinics, FM clinic and SS clinic, in 1 year. The duration was 260 days.

**Additional file 2: Table S2.** The details of the checklist according to the 2015 Thai Hypertension Guidelines.

## Data Availability

Data set are available from the corresponding author on request.

## Abbreviations

BMI: body mass index
BP: blood pressure
Cr: creatinine
CPG: clinical practice guidelines
CV: cardiovascular
FM: family medicine
GFR: glomerular filtration rate
HDL-C: high-density lipoprotein cholesterol
LDL-C: low-density lipoprotein cholesterol
PCM: patient-centered medicine
PR: pulse rate
TC: total cholesterol
TOD: target organ damage
WC: waist circumference
