# Supplementary material for: Incorporating the patient-centered approach into clinical practice helps improve quality of care in cases of hypertension: a retrospective cohort study
Source: BMC Fam Pract. 2020 Jun 12;21:108. doi: 10.1186/s12875-020-01183-0 (PMC7293111; doi:10.1186/s12875-020-01183-0)
Supplement: Supplementary file 1 — Additional file 1: Table S1. The demographic data of two primary outpatient department clinics, FM clinic and SS clinic, in 1 year. The duration was 260 days. [file 12875_2020_1183_MOESM1_ESM.docx]

**Supplementary Table 1**

**Table S1.** Demographic data of two primary outpatient department clinics in one year (Duration 260 days)

| Demographic data | FM clinic | SS clinic |
| --- | --- | --- |
| Aim of contribution | Training and service  (for medical students and residents) | Service |
| Patient’s coverage for use of the health care service | Civil servant benefit scheme and direct payment | Social security scheme |
| Total visits per year  Total visits per day | 29,496  113 | 58,412  225 |
| Total visits about hypertension per year  Total visits about hypertension per day | 8,565  33 | 15,838  61 |
| % of hypertension patients to all patients | 29.04 | 27.11 |
| Number of service doctors per day | 2-9 | 4-6 |
| - Regular service staff | 1-4 | 1-2 |
| - Fellowships | - | 0-1 |
| - Residents | 1-5 | 2 |
| - First year internist | - | 1 |
| Physician mean age (SD) | 38.26 (15.98) | 31.68 (12.49) |
