# Supplementary material for: Incorporating the patient-centered approach into clinical practice helps improve quality of care in cases of hypertension: a retrospective cohort study
Source: BMC Fam Pract. 2020 Jun 12;21:108. doi: 10.1186/s12875-020-01183-0 (PMC7293111; doi:10.1186/s12875-020-01183-0)
Supplement: Supplementary file 2 — Additional file 2: Table S2. The details of the checklist according to the 2015 Thai Hypertension Guidelines. [file 12875_2020_1183_MOESM2_ESM.docx]

**Supplementary Table 2**

**Table S2.** Details of the Checklist According to the 2015 Thai Hypertension Guidelines

| Topics | Details | Period of assessment |
| --- | --- | --- |
| **History taking** | *Any record of the following details was considered as “performed”* | |
| - History of hypertension | Symptoms, duration, type of anti-hypertensive drug, blood pressure control, or side effect from anti-hypertensive medication | One year |
| - Family history of hypertension, n | Family history of hypertension | Since first diagnosed |
| - Risk factors | Smoking, alcohol drinking, sedentary lifestyle, high salt diet, diabetic mellitus, dyslipidemia, or obstructive sleep apnea | One year |
| - Symptoms suggested TOD | Palpitation, dyspnea, chest pain, numbness, weakness on extremities, blurred vision, headache, dizziness, leg edema, or intermittent Claudication | One year |
| - Assessment for secondary hypertension | Blood pressure swinging, headache, palpitation, sweating, weakness, back pain with abnormal urination, or drug use such as oral contraceptive pills, cocaine, amphetamine, steroid, NSAIDs, or pseudoephedrine | Since first diagnosed |
| **Physical examination** | *Any record of the following details was considered as “performed”* | |
| - BP and PR measurement | BP and PR measurement | One year |
| - BMI calculation and WC measurement | BMI calculation or WC measurement | One year |
| - TOD and cardiovascular disease | TOD and cardiovascular disease examination including left ventricular hypertension (murmur, heaving, thrill, or shifting apex), stroke or transient ischemic attack (neurological examination), vascular disease (auscultation of bruits, lower extremity edema, pulse, or leg blood pressure), or retinopathy (optic fundi) | One year |
| - Evidence of secondary hypertension | Evidence of secondary hypertensive examination including thyroid disorders (bradycardia/tachycardia), Cushing’s syndrome (buffalo hump, central obesity, moon face, or striae), coarctation of the aorta (arm to leg systolic blood pressure difference > 20 mmHg, delayed or absent femoral pulses, or murmur), pheochromocytoma (flushing, orthostatic hypotension, or sweating), or renal artery stenosis (renal bruise) | Since first diagnosed |
| **Laboratory investigation** | *At least one set of lab results shown in details was considered as “performed”* | |
| - Fasting plasma glucose | Fasting plasma glucose | One year |
| - Serum TC, HDL-C, LDL-C, triglyceride | Serum TC, HDL-C, LDL-C, or triglyceride | One year |
| - Serum electrolytes, Cr, GFR calculation | Serum electrolytes, Cr, or GFR calculation | One year |
| - Hemoglobin or hematocrit | Hemoglobin or hematocrit | One year |
| - Urinalysis | Urinalysis | One year |
| - Electrocardiography | Electrocardiography | One year |
| - CV risk assessment | Record of percentage of estimated 10-year risk | One year |
| **Treatment by lifestyle modification** | *Any record of the following details was considered as “performed”* | |
| - Weight reduction | Any record about suggestion on weight management | One year |
| - Appropriate exercise | Any record of exercise advice including moderate intensity exercise, duration of at least 30 minutes per day, or frequency 5 days per month | One year |
| - Dietary approach | Any record of dietary advice including low fat diet, high fiber diet, sugar control diet, types of fruit and vegetable, or low salt diet | One year |
| - Limit alcohol intake for drinkers | Evidence of giving of advice for drinkers | One year |
| - Smoking cessation for smokers | Evidence of giving of advice for smokers | One year |
| **Treatment by anti-hypertensive medication** (*Considered “appropriate” if …*) | | |
| - Starting with appropriate dosage of medication | - Start with one type of medication if SBP 140-160 mmHg DBP 90-100 mmHg and low CV risk  *Or*  - Start with two types of medication if SBP >160 mmHg DBP >100 mmHg or high CV risk | At the first visit diagnosing hypertension |
| - Use of appropriate class at the start | Starting with one of the 4 classes includes Thiazide-type diuretic, CCBs, ACEIs, or ARBs | At the first visit on prescription of medication |
| - Appropriate choice of medication for specific conditions | Recommended classes were chosen for patients with specific conditions including older age, cardiovascular disease or high risk, cerebrovascular disease, diabetic mellitus, chronic kidney disease, and female or pregnancy. | Since first diagnosed |
| - Incorrect use of ACEIs together with ARBs | Never use ACEIs together with ARBs (among patients prescribed with more than one type of medication) | One year |
| - Prescription of fixed-dose combinations | Choosing fixed-dose combinations instead of using divided pills (among patients who had ever used more than one type of medication) | One year |
| - Adjustment of dosage of medication when blood pressure goal was not achieved | Increase dose or add additional medication ever uncontrolled in any visit | One year |
